# Spatial heterogeneity and scale‐dependent habitat selection for two sympatric raptors in mixed‐grass prairie

**DOI:** 10.1002/ece3.3182

**Published:** 2017-07-15

**Authors:** Fidelis Akunke Atuo, Timothy John O'Connell

**Affiliations:** ^1^ Department of Natural Resource Ecology and Management Oklahoma State University Stillwater OK USA

**Keywords:** competition, heterogeneity, landscape, niche breadth, Northern Harrier, predator, Red‐tailed hawk, resource segregation

## Abstract

Sympatric predators are predicted to partition resources, especially under conditions of food limitation. Spatial heterogeneity that influences prey availability might play an important role in the scales at which potential competitors select habitat. We assessed potential mechanisms for coexistence by examining the role of heterogeneity in resource partitioning between sympatric raptors overwintering in the southern Great Plains. We conducted surveys for wintering Red‐tailed hawk (*Buteo jamaicensis*) and Northern Harrier (*Circus cyanea*) at two state wildlife management areas in Oklahoma, USA. We used information from repeated distance sampling to project use locations in a GIS. We applied resource selection functions to model habitat selection at three scales and analyzed for niche partitioning using the outlying mean index. Habitat selection of the two predators was mediated by spatial heterogeneity. The two predators demonstrated significant fine‐scale discrimination in habitat selection in homogeneous landscapes, but were more sympatric in heterogeneous landscapes. Red‐tailed hawk used a variety of cover types in heterogeneous landscapes but specialized on riparian forest in homogeneous landscapes. Northern Harrier specialized on upland grasslands in homogeneous landscapes but selected more cover types in heterogeneous landscapes. Our study supports the growing body of evidence that landscapes can affect animal behaviors. In the system we studied, larger patches of primary land cover types were associated with greater allopatry in habitat selection between two potentially competing predators. Heterogeneity within the scale of raptor home ranges was associated with greater sympatry in use and less specialization in land cover types selected.

## 1 INTRODUCTION

Understanding the effects of spatial heterogeneity on interference, coexistence, niche separation, and differential habitat selection among species is a key concept in community ecology (Kalcounis‐Rüppell & Millar, 2002; MacArthur, 1972). Studies of ecological segregation or diet overlap among sympatric species often seek to explain how species or populations might differ in their use of limited resources (González‐Solís, Oro, Jover, Ruiz, & Pedrocchi, 1997; Traba et al., 2013). For example, MacArthur (1958) found that five closely related species of *Setophaga* (nee *Dendroica*) warblers coexisted in boreal forest by foraging in different portions of trees. Although these warblers overlapped broadly at home range scales, each specialized behaviorally to partition resources at the scale of individual trees.

The ability of predators to sight, pursue, capture, and consume prey is often influenced by the structural complexity of the landscape (Gorini et al., 2012). Spatial heterogeneity can lead to an increase or a decrease in hunting success depending on the specific behavioral characteristics of the predator (Oliver, Luque‐Larena, & Lambin, 2009). In systems with potentially competing predators, resource partitioning is expected for strongly preferred and or limited resources. Where two predator species select similar prey, differences in habitat selection can be sufficient to reduce competition through niche partitioning.

Habitat selection and niche segregation/overlap are hierarchical processes in which the patterns that are detected are frequently dependent on scale of the study (Kotliar & Wiens, 1990; Morris, 2003). At broad spatial scales, multiple species overlap while segregation is likely to occur at finer scales (Soto & Palomares, 2015; Traba et al., 2013). Habitat selection can also vary temporally with attendant consequences for competing species. For example, seasonally low resource availability during winter increases both interspecific and intraspecific competition in temperate environments (Diggs, Marra, & Cooper, 2011; Pulliam & Mills, 1977). During these constrained periods, competition is high and we expect to see broad overlap in habitat selection among species with similar resource requirements.

Temperate grasslands of the United States support multiple species of diurnal raptors, with annual residents, breeding migrants, and wintering migrants represented. During winter when energetic demands are high, population densities of Red‐tailed hawk (*Buteo jamaicensis*) and Northern Harrier (*Circus cyaneus*) reach their annual peaks, and broadscale sympatry in habitat use can presumably lead to competition. Both species are opportunistic predators of small mammals, birds, reptiles, and amphibians (Collopy & Bildstein, 1987; Preston, 1990; Preston & Beane, 2000; Redpath & Thirgood, 1999; Turner et al., 2014). The two raptors differ in their behavior and hunting strategy, but will often rely on similar prey (primarily the Hispid Cotton Rat, *Sigmodon hispidus*) during winter (Behney, Boal, Whitlaw, & Lucia, 2011; Lish, 2015; Turner et al., 2014).

In this study, we compared habitat selection of Red‐tailed hawks and Northern Harriers overwintering in the Great Plains in two landscapes that differed in land cover heterogeneity. We tested two primary hypotheses: (1) Sympatry in habitat selection will be greater where heterogeneity is higher, and (2) fine‐scale habitat selection will be more sensitive to heterogeneity than selection at broader scales.

## 2 METHOD

### 2.1 Study sites

**Figure 1:**
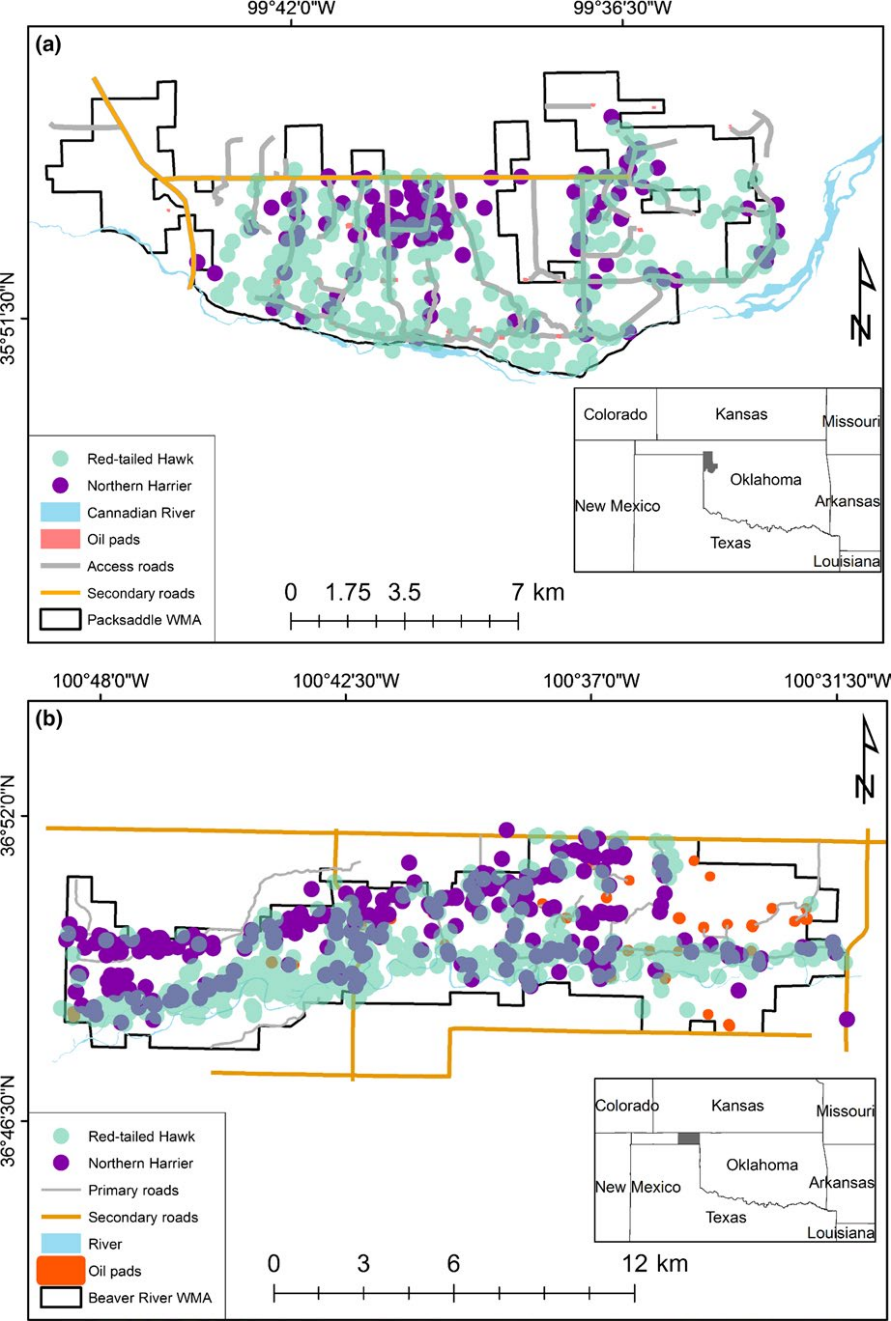
Occurrence points of Red‐tailed Hawk (green) and Northern Harrier (purple) at Packsaddle (a) and Beaver River (b) Wildlife Management Areas (WMAs) in Oklahoma, USA, 2013–2015

Our study was conducted within two wildlife management areas (WMAs) managed by the Oklahoma Department of Wildlife Conservation in western Oklahoma, USA. Packsaddle WMA (Fig. 1a) covers ~6,475 ha with an elevation 579–762 m asl. The dominant vegetation is shinnery oak (*Quercus havardii*) mixed with codominant grasses and forbs. Shinnery oak thrives on sandy soils where it readily resprouts following fire and spreads clonally through rhizomes. Across broad areas of Oklahoma and Texas, shinnery oak can produce extensive stands of dwarf trees approximately 1 m tall. Interspersed among those stands can also occur isolated mottes of taller (e.g., 3–6 m) oak trees that are typically hybrids of shinnery and post oak (*Quercus stellata*). Detailed information on climate, soils, and vegetation community available in the study site has been described (DeMaso, Peoples, Cox, & Parry, 1997; Hall, 2015). Beaver River WMA (Fig. 1b) is ~7,163 ha in area, consisting of a mixture of upland, floodplain, and river bottom. Vegetation in uplands is predominantly sagebrush (*Artemisia tridentate*) and buffalograss (*Bouteloua dactyloides*) interspersed with sand plum (*Prunus angustifolia*) thickets and gently rolling sandhills. The floodplain portion of the WMA is comprised mostly of grasses mixed with cottonwood (*Populus deltoides*), hackberry (*Celtis occidentalis*), and American elm (*Ulmus americana*). The river bottom (generally dry riverbed) is woody vegetation consisting of sand plum thickets and salt cedar (*Tamarix* spp).

### 2.2 Predator surveys

We conducted November–May surveys for Red‐tailed hawk and Northern Harrier, 2013–2015. We surveyed 30 line transects (14 at Packsaddle 16 at Beaver River) ranging 2–9 km in length. The length for all transects was 49.42 km for Packsaddle and 58.98 km for Beaver River. All but two transects at Packsaddle WMA were placed along existing trails (Fuller & Mosher, 1987). We separated transect by a distance of <1,000 m to reduce the chance of counting an individual more than once per survey. We surveyed each transect at least twice a month (260 total surveys) using a 4‐wheel truck driven at a speed of 20–30 km/h. During each survey, an observer scanned for raptors a distance of approximately 500 m on either side of the transect line. We georeferenced (Garmin Montana 650TM GPS) each detection at the point of observation and estimated distance from the transect line using a laser rangefinder and angle of observation from the observer using an azimuth compass. To develop a spatially explicit model of predator distribution, we plotted each detection point to the point of occurrence in time using the approach described in Atuo and O'Connell (2017).

### 2.3 Vegetation classification

We obtained Geo‐Eye images for Beaver River and Packsaddle WMA through the Oklahoma Department of Wildlife Conservation. Two Geo‐Eye images were acquired in July of 2014 with approximately 2 m spatial resolution in the visible (panchromatic) spectrum. The land cover maps for 2014 were considered adequate for our vegetation classification, as there were no significant landscape changes in the year before or after 2014.

To obtain vegetation and landscape attributes for each study site, we performed a supervised classification on the preprocessed image using the maximum likelihood algorithm. Identified land cover classes at Packsaddle included upland forest, riparian forest, grassland, bare ground, water, sparse vegetation cover, and oil installation. At Beaver River, identified land cover classes were upland shrub, riparian forest, grassland, sparse vegetation cover, and bare ground.

To model habitat selection, we extracted land cover information centered at each use (offset point) location of Red‐tailed hawk and Northern Harrier at two spatial scales. First, we plotted raptor occurrence points in a GIS and created 300 m radius buffers centered at each occurrence point to represent a fine scale (28 ha) that might provide immediate foraging resources (Amar & Redpath, 2005; Stout, 2004). We then extracted the proportion of pixels representing each vegetation cover type. At a broader home range scale, we collected environmental variables that were summarized into three groups (vegetation, topographical, distance‐related covariates). To categorize and quantify vegetation, we plotted buffers of 1,000 m radius (314 ha) around occurrence points to represent 50%–95% home range sizes estimated for the two species (Arroyo, Leckie, Amar, Mccluskie, & Redpath, 2014; Stout, Temple, & Cary, 2006) and extracted the proportion of each vegetation cover type. For distance to key landscape features, we calculated Euclidean distances from used and available (randomly selected) locations to the closest layer paved roads, access roads, rivers, and oil pads. In addition to land cover, distance‐based variables can be important because species might select areas based on their proximity to resources without actually selecting the habitat or landscape class itself. We obtained topographical variables (i.e., aspect, slope, and elevation) from the digital elevation model (DEM). DEM data were collected from the United States Geological Survey (USGS) data portal at 1/3 arc‐seconds resolution. We linearized aspect into two continuous variables of northness (the cosine of aspect) and eastness (the sine of aspect) (Domínguez & Dirzo, 1995). To compare resource use to availability, we generated random (available) locations equaling the number of use locations for each species using the random number generator tool in ArcGIS 10.2.2 (Environmental Systems Research Institute Inc., Redlands, CA, USA). Available locations were constrained within 500 m radius of each transect consistent with the detection distance equaling the number of use locations for each species and extracted vegetation variables. We quantified the spatial heterogeneity at each study site within three concentric buffers of 50 m, 100 m, and 200 m radii centered around 100 random points generated in a GIS. Multiple scale buffers were necessary due to expected scale‐specific responses of our study organisms. Buffer sizes were selected based on previous studies (Stohlgren, 2007) that have shown them well suited for quantifying vegetation diversity. Within each buffer, we extracted the number of pixels that represented each vegetation class and computed Shannon diversity index of all buffered areas (Fahrig et al., 2011).

### 2.4 Data analyses

We performed our analyses in three major steps. First, we performed distance analyses to estimate species abundance and detection probabilities. Second, we analyzed habitat selection of each species in ecological space. Third, we performed a discriminant analysis of habitat used to test for spatial segregation or overlap in the ecological niches of the two species.

At each study site, we estimated a distance detection function for each species by computing detection probabilities using the multiple‐covariate distance sampling approach (Buckland, Rexstad, Marques, & Oedekoven, 2015; Marques, Thomas, Fancy, Buckland, & Handel, 2007). The detection function model estimates detection probabilities with increasing distances from transect lines. We compared a suite of a priori candidate models including half‐normal, hazard‐rate, and uniform function keys with cosine adjustment terms. We included different covariates (time of the day, month of survey, observer ID, and their interactions) to increase the explanatory power of our models. We treated all covariates as factors including time of day which was categorized into five time intervals: Morning (0700–0900), late morning (0901–1100), midday (1101–1300), afternoon (1301–1500), and later afternoon (>1500 hr CST). We ranked models using the Akaike Information Criterion (AIC) and collected detection probabilities based on the best competing models within a ΔAIC value <2. For both species, we estimated detection corrected density based on the best model for the detection function. To account for the effects of multiple visits on density estimates, we computed survey efforts as the number of survey events for each transect multiple by the transect length. We performed all distance analyses using program *Distance 6.2*.

We characterized habitat selection by comparing environmental variables collected at occurrence points to those collected from random points. We used the generalized mixed linear model (GLMM) approach with binomial error structures to estimate habitat selection at both study scales. At each level, we included year as a random effect to account for variation in raptor abundance across sampling duration. Fixed effects were defined and varied depending on the study site and scale of analysis. At the broadscale, fixed effects included vegetation extracted from land cover maps, topographical variables extracted from the DEM, and distance‐related covariates measured as Euclidean distances to identified landscape features. We performed a Pearson correlation on all variables at each scale and removed variables that were redundant (|*r*| > .7).

To reduce model complexity, we employed a two‐step approach to build habitat selection models. First, we identified the environmental variables associated with Red‐tailed hawk and Northern Harrier habitat selection in our study region based on previous studies (McConnell, O'Connell, & Leslie, 2008; Preston, 1980) and developed models based on these known variables. Second, using these parsimonious models as bases, we developed a set of a priori candidate models by examining the additive and interactive roles of additional covariates and a random effect of year. We then ranked and averaged all candidate models according to their Akaike's information criterion values adjusted for small sample size (AICc; Burnham & Anderson, 2002) using the MuMIn package (Barton, 2016). We considered competing models within a ΔAICc < 2 as important in explaining habitat selection providing they were not variants of the best model plus one uninformative parameter (Arnold, 2010). We evaluated model‐averaged estimates for variables of interest in competing models and calculated unconditional standard errors and 95% confidence limits (Arnold, 2010; Burnham and Anderson, 2002). Prior to statistical analysis, we standardized all environmental variables to a mean of 0 and a standard deviation of 1 to improve variable interpretation.

To estimate ecological space filled by Red‐tailed hawk and Northern Harrier, we performed an Outlying Mean Index analysis (OMI) (Dolédec, Chessel, & Gimaret‐Carpentier, 2000) using the R package ADE4 (Dray & Dufour, 2007). The OMI, or species marginality analysis, is a multivariate analysis technique (based on principle components analysis) that estimates the distance between mean habitat conditions used by a species (species centroid) and the mean habitat conditions that exist in the study landscape. An OMI analysis places species along a habitat gradient (niche hyperspace) based on their mean abundances. The hyperspace represents the theoretical niche of a species that can tolerate all habitat conditions available in the study area (i.e., a species that is distributed uniformly across the landscape). Marginality is a measure of how far a species occurs away from the origin of the niche hyperspace. Hence, the marginality of a species depends on its deviation from the origin of the niche hyperspace. Species with higher marginality scores represent a deviation from the mean conditions available in the landscape. The OMI analysis also calculates a species’ tolerance (i.e., species niche breadth). Species with higher tolerance values (generalists) can occupy varying habitat conditions while those with low tolerance values (specialists) are limited in their habitat use. We estimated niche space parameters (marginality and tolerance) for Red‐tailed hawk and the Northern Harrier at the microhabitat scale only because GLMM analyses demonstrated significant overlap at higher scales. We determined significance of the OMI based on a Monte Carlo simulation of 10,000 random permutation values of species marginalities.

## 3 RESULTS

We completed a total survey effort (total transect length × number of visits) of 472 km at Beaver River and accumulated 963 detections of Red‐tailed hawk and 681 detections of Northern Harrier. At Packsaddle, we surveyed a total of 395 km and recorded 558 detections of Red‐tailed hawk and 241 detections of Northern Harrier. The best detection models for Red‐tailed hawk detection included the variables *observer* and *time of day* at Packsaddle (see Table S1), and *observer* and *month* at Beaver River WMA (Table S2). At Packsaddle WMA, the best model for Northern Harrier detection included month of survey and observer (Table S2). Harrier detection at Beaver River was best explained by month of survey (Table S2). At both sites, the Hazard‐rate key function provided the strongest support for detection (Tables S1 and S2).

Generally, mean detection probabilities were higher at Beaver River than Packsaddle WMA (Fig. S1) and higher for Red‐tailed hawk than for Northern Harrier (Fig. S1). Estimated density of Red‐tailed hawk was slightly higher at Beaver River 1.77 ± 0.01 (95% CI: 1.64–1.90)/100 ha than at Packsaddle 1.37 ± 0.01 (95% CI: 1.20–1.47)/100 ha. Northern Harrier density was 2.470 ± 0.002 (95% CI: 2.240–2.720)/100 ha at Beaver River and 2.22 ± 0.02 (95% CI: 1.87–2.64)/100 ha at Packsaddle WMA.

### 3.1 Landscape heterogeneity

At all scales, land cover heterogeneity was higher (*p* < .001) at Packsaddle WMA. Mean (±*SE*) Shannon Diversity Index for Packsaddle was 1.05 ± 0.03 (50 m), 1.17 ± 0.03 (100 m), and 1.22 ± 0.02 (200 m). At Beaver River, corresponding mean (±*SE*) Shannon Diversity Index values were 0.91 ± 0.03 (50 m), 0.98 ± 0.03 (100 m), and 1.01 ± 0.03 (200 m).

### 3.2 Habitat selection at Beaver River

**Figure 2:**
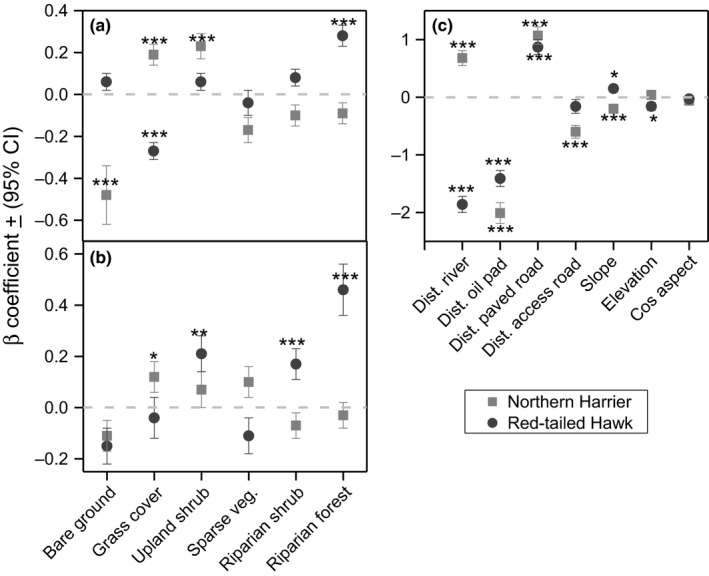
Beta coefficients of (β + 95% CI) for habitat selection by Northern Harrier and Red‐tailed Hawk at Beaver River Wildlife Management Area in Oklahoma, USA, 2013–2015. Coefficients were collected from a resource selection function of each species at >28 ha (a) and >201 ha (b). Additional coefficients for topographical and distance‐related covariates are presented (c). The level of significance is donated by asterisk where *p < .05; **p < .01; and ***p < .001

At Beaver River, the most approximating of 27 models evaluated for Northern Harrier habitat selection included grass cover, bare ground, upland shrub cover, and sparse vegetation. The second model, which was the only other model within ΔAICc < 2, was considered as a nested version of the approximating model with one additional uninformative covariates (Table S3; Arnold, 2010). This model indicated that harriers selected for grassland and upland shrub cover while avoiding bare ground, sparse vegetation, and riparian woodland (Table S3). For Red‐tailed hawk selection, we evaluated 29 models. The best‐supported models showed selection for riparian forest, upland shrub cover, bare ground, and avoidance of grass cover. Unconditional parameter estimates based on model averaging indicated that all four variables in the approximation model were significant (*p* < .05) in informing Northern Harrier selection while selection for riparian forest cover and avoidance of grass cover were the only significant (*p* < .05) variables for Red‐tailed hawk selection at Beaver River (Fig. 2). At broadscale, importance variables for Northern Harrier selection included grass cover, upland shrub cover, and areas with sparse vegetation cover (Table S3). Riparian forest was the only variable within ΔAICc < 2 that was avoided. The best approximating model for Red‐tailed hawk selection suggested selection for riparian forest, riparian shrub, and upland shrub cover (Fig. 2) while avoiding bare areas, grass cover, and areas with sparse vegetation cover. Based on an unconditional parameter estimates, grass cover was the most significant (*p* < .05) variable for harrier selection while riparian forest, riparian shrub, and upland shrub cover were the most significant variables for Red‐tailed hawk selection at Beaver River WMA (Fig. 2).

### 3.3 Habitat selection at Packsaddle

At fine scale, we interpreted two competitive models from 32 a priori model sets as best approximating models for Red‐tailed hawk selection at Packsaddle WMA. They included the models with riparian forest as its only fixed effect, and the model with riparian forest and oil pads (Table S4). Generally, Red‐tailed hawk selection was in favor of riparian forest, upland forest, and sparse vegetation while oil pads, bare ground, and grass cover were avoided (Table S4). From the 47 models evaluated for Northern Harriers, selection was in favor of grass cover, upland forest, and an interaction between upland forest and grass cover. Bare ground, riparian forest, and areas with sparse vegetation were avoided (Fig. S4). Unconditional parameter estimates based on model averaging suggested that riparian forest was the most significant (*p* < .05) variable for Red‐tailed hawk selection while grass cover and upland forest were the most significant (*p* < .05) variables for Northern Harrier selection at Packsaddle WMA (Fig. S4).

**Figure 3:**
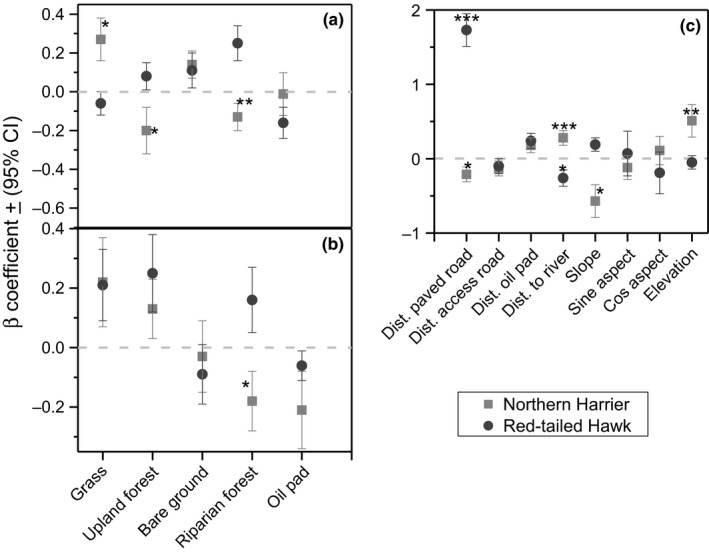
Beta coefficients of (β + % CI) for habitat selection by Northern Harrier and Red‐tailed Hawk at Packsaddle Wildlife Management Area in Oklahoma, USA, 2013–2015. Coefficients were collected from a resource selection function of each species at >28 ha (a) and >201 ha (b). Additional coefficients for topographical and distance‐related covariates are presented (c). The level of significance is donated by asterisk where *p < .05; **p < .01; and ***p < .001

At broadscale, we evaluated 18 models for Red‐tailed hawk selection. The best approximating model shows positive selection for grass cover, riparian forest, and upland forest. Of the 29 models evaluated for Northern Harrier selection, we interpreted two competing models with ΔAICc < 2 as the best approximating models (Table S4). The two models together with their nested versions suggested that harrier selected for grass cover, bare ground, upland forest, and areas with sparse vegetation cover. They however avoided riparian forest. Conditional averaging did not identify any significant difference for any of the variables evaluated for the Red‐tailed hawk (Fig. 3). Meanwhile similar estimates for Northern Harrier showed significant selection for grass cover, upland forest (Fig. 3).

### 3.4 Selection in relation to distance from major landscape features

Global models with four distance‐related covariates were in each case interpreted as best approximating models for Northern Harrier, and Red‐tailed hawk selection at Beaver River (Table S3). All four measured covariates for Northern Harrier, and three covariates for Red‐tailed hawk at Beaver River differed (*p* < .05) from random (Fig. 2). Both species selected areas that were away from paved roads and oil pads but in proximity of access (primary) roads (Fig. 2). However, the likelihood of harrier selection decreased with decreasing distances to riparian forest while Red‐tailed hawk selected areas that were in proximity to riparian forest (Fig. 2). At Packsaddle, the best approximating model for habitat selection by Red‐tailed hawk included distance to paved roads as its only fixed effect (Table S4). The likelihood of Red‐tailed hawk selection increased with increasing distance to riparian forest, paved roads, and oil pads (Fig. 3). Unconditional model averaging suggested that distances to paved roads and river were important variables informing harrier selection (Table S4). Overall, the likelihood of harrier selection increased farther from paved roads and riparian forest (Fig. 3).

### 3.5 Selection in relation to topographical variables

We evaluated 27 models for each species at each site to understand the effects of topographical variables on selection (Table S4). At Packsaddle WMA, Northern Harrier habitat selection increased as elevation increased and decrease with increasing slope (Fig. 2) while Red‐tailed hawk selection decreased with increasing slope (Fig. 3). Unconditional model averaging suggested that slope and elevation were the most important variables for Northern Harrier selection, whereas slope was the most significant variable for Red‐tailed hawk selection (Fig. 3).

At Beaver River WMA, the best approximating model of 27 models for Northern Harrier selection showed a decreasing likelihood of harrier selection with increasing decree of slope (Fig. 2). Two competing models were considered as the best approximating models for Red‐tailed hawk selection (Table S3). The likelihood of Red‐tailed hawk selection increased with increasing degree of slope and decreased with elevation (Fig. 2). Based on conditional model averaging, slope and elevation (both in its linear and quadratic forms) were the most significant (*p* < .05) variables for Red‐tailed hawk selection whereas decreasing degree of slope was the most significant variable for Northern Harrier selection at Beaver River WMA (Fig. 2).

### 3.6 Niche overlap

**Figure 4:**
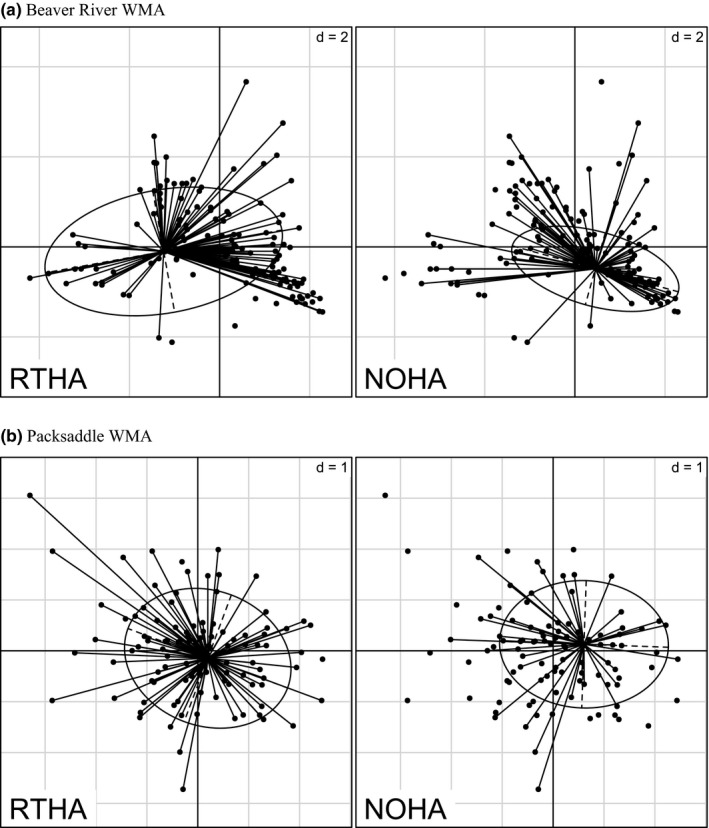
Outlying Mean Index analysis of Red‐tailed Hawk (RTHA) and Northern Harrier (NOHA) at Beaver River (a) and Packsaddle (b) Wildlife Management Areas (WMAs) in Oklahoma, USA, 2013–2015. The points represent weighted positions of each species in ecological niche space. The figure represents the ecological position of the two species in the *n*‐dimensional hypervolume. The ellipses show the 95% confidence interval around the mean

**Figure 5:**
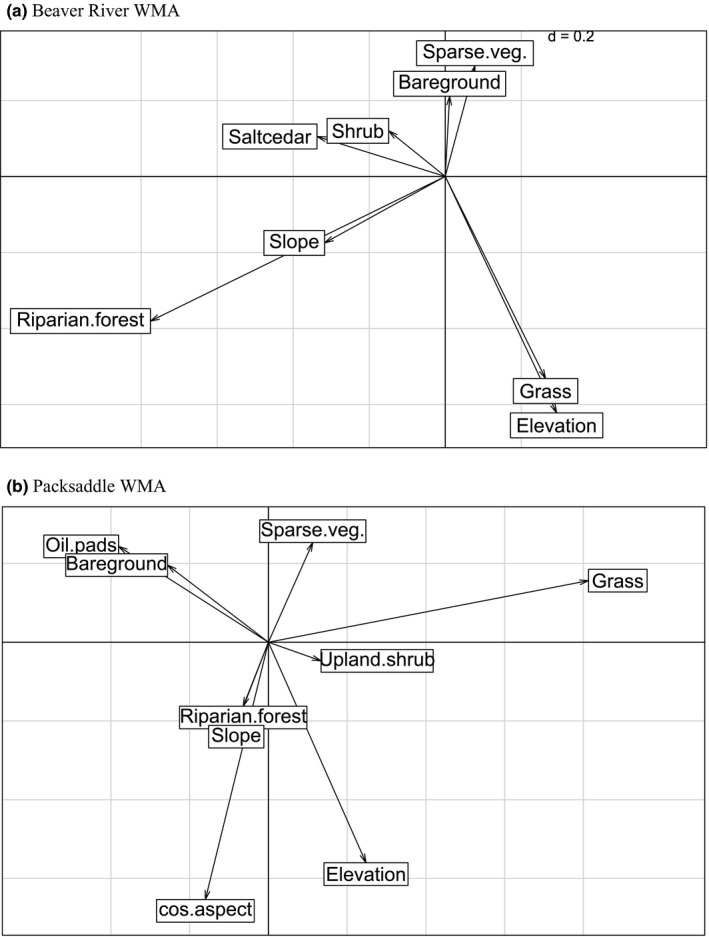
Canonical weights of environmental variables at Beaver River (a) and Packsaddle (b) Wildlife Management Areas (WMAs) in Oklahoma, USA, 2013–2015. The figure represents the contribution of environmental variables to the definition of niche parameters of species in the Outlying Mean Index analysis. The length of the arrow describes the relative importance of each variable, and the direction of the arrow indicates among‐variable correlations

The Outlying Mean Index analysis illustrated significant (*p *< .005) segregation at Beaver River but not at Packsaddle. At Beaver River, the Monte‐Carlo randomization test showed that both marginality and tolerance values were significantly different from 0 (i.e., the reference point of the total niche space) for Red‐tailed hawk (*p* < .001) and Northern Harrier (*p* = .023, Table S5). At Packsaddle, marginality and tolerance values for both species did not differ from 0 (*p* > .05, Table S5). Ordination diagrams show that the two species occupied different axes and were separated from each other in environmental space (Fig. 4). Our ordination diagrams for Packsaddle suggested a positive association of Red‐tailed hawk with both sides of the second axis (Fig. 4). At Beaver River WMA, the first OMI‐axis was driven by altitude and the proportion of grassland at the positive end of the gradient and by sparse vegetation cover at the opposite end. The second axis was positively influenced by forest cover, riparian shrub, and cosine of aspect. At the negative end of the gradient were shrub and the sine of aspect. The presence of the Red‐tailed hawk was best discriminated by forest cover, riparian shrub, and shrub cover whereas Northern Harrier was more positively associated with higher altitude and by the proportion of grassland (Fig. 5a). The first OMI‐axis at Packsaddle was positively influenced by elevation and negatively by grass cover, while the second axis was driven by the cosine of aspect, slope, and riparian forest at the positive end by the proportion of bare ground and oil pads at the negative end. Accordingly, Red‐tailed hawk was positively associated with riparian and upland woody cover while Northern Harrier demonstrated positive affinity for grassland (Fig. 5b).

## 4 DISCUSSION

Red‐tailed hawk and Northern Harrier are sympatric over much of their winter distribution in temperate North America (e.g., Arkansas: Preston, 1990). Both species hunt primarily from grasslands and other early successional environments and generally seek small mammal prey (Baker & Brooks, 1981; Bildstein, 1987; Orians & Kuhlman, 1956; Redpath & Thirgood, 1999). High densities of both species overwintering in Great Plains’ grasslands could lead to competition, especially considering the importance of one species, Hispid Cotton Rat, in the diets of wintering raptors (Lish, 2015) in the southern Great Plains. We found the two raptor species to be broadly sympatric at the two study sites during winter, with several dozen individuals of each species co‐occurring at scales of several thousand hectares.

Red‐tailed hawk and Northern Harrier differ, however, in their typical hunting behaviors, and this might help them to partition resources and reduce competition at finer scales. Red‐tailed hawk is primarily a sit‐and‐wait predator that occupies elevated perches for long periods before darting out to catch its prey (Lish, 2015). Thus, the effective hunting area for a Red‐tailed hawk is likely limited by the juxtaposition of favorable perches and suitable grasslands to provide prey. Previous studies have indicated selection for riparian and upland woody vegetation for perches of hunting Red‐tailed hawks (Garner & Bednarz, 2000) and these were selected even when anthropogenic perches such as utility poles were available (Bobowski, Rolland, & Risch, 2014). In contrast, Northern Harrier hunts while flying, generally coursing back and forth above the grasslands and pouncing when it detects potential prey in the grass (MacWhirter & Bildstein, 1996; Simmons, 2000). Harriers are not limited by trees or other elevated perches (Littlefield, Johnson, & Brush, 2005). In a broad grassland complex therefore, we might expect the two predators to be sympatric at broadscales, but to partition fine‐scale habitat use such that Red‐tailed hawk tends to occupy areas in proximity to forested zones. Northern Harriers, however, would be expected to occupy grassland patches in open areas farther away from trees. Fine‐scale segregation that describes a tendency toward edge (Red‐tailed hawk) and interior (Northern Harriers) of the same grassland patches could be sufficient to partition hunting areas and permit sympatry at broader scales.

Our results confirmed segregation in habitat use between the two raptor species, but the scale and degree was associated with land cover heterogeneity. For land cover types, Shannon diversity index scores were higher at Packsaddle than at Beaver River WMA at all three scales that we tested: 0.79 ha, 3.14 ha, and 12.56 ha. We interpreted this result as Packsaddle supporting a more heterogeneous landscape than did Beaver River WMA, and at scales within the home range and foraging area expectations for both Northern Harrier and Red‐tailed hawk.

Compared to Packsaddle, dominant land cover types at Beaver River WMA occurred in larger and more discrete patches. One result of this that could have influenced habitat selection was that trees at Beaver River were largely confined to the riparian zone while grass and low shrub cover dominated the uplands. This is a common pattern of land cover structure in the Great Plains (Liu et al., 2013). Red‐tailed hawks hunting from perch trees would thus be more likely to be detected in proximity to the riparian corridor while Northern Harriers were free to hunt the grasslands regardless of distance to the riparian corridor. At Packsaddle WMA, however, trees were not restricted to the riparian corridor (DeMaso et al., 1997). The uplands at Packsaddle were characterized by many scattered clonal growths of hybrid shinnery oak growing in a largely grass matrix. These taller oak mottes in an upland grassland matrix provided convenient perches for Red‐tailed hawks so they were not restricted to the riparian zone at Packsaddle as at Beaver River WMA. These anecdotal impressions of habitat use were supported by the OMI analysis that confirmed first that both raptors deviated in habitat selection from average conditions at Beaver River, where Northern Harrier selected upland grass cover and avoided the riparian zone and Red‐tailed hawk selected the riparian zone and avoided upland grass cover (Figs 4 and 5). In contrast, neither species deviated from average condition at Packsaddle WMA where both species selected available cover in proportion to its abundance (Figs 4 and 5).

With respect to our hypotheses of segregation and scale for Red‐tailed hawk and Northern Harrier, our results confirmed greater sympatry (i.e., less segregation) in the more heterogeneous landscape at Packsaddle WMA. There, we found relatively weak statistical support for segregation in selection of grass cover, upland forest, and riparian forest. In contrast, statistical support for segregation at the more homogeneous Beaver River WMA was stronger and included more variables (Figs 2 and 3). At both WMAs, there was also better support for segregation at the finer scale (28 ha) than over a broader area (314 ha). Even at scales <28 ha, both predators were more likely to hunt the same patches at Packsaddle than at Beaver River WMA. These two predators provide a case of independent corroboration for previous work (e.g., Holt, 1984) predicting that in heterogeneous landscapes, competing species are less likely to show distinct spatial segregation. Thus, the composition and structure of the landscapes affected habitat selection and behavior of the predators, illustrating an example of landscape function potentially shaped by landscape form.

Predators’ distribution and habitat selection are often explained by hunting successes associated with certain cover types, and the presence of competitors (Gorini et al., 2012). However, spatial heterogeneity can modify hunting abilities, strategies, and efficiencies across the landscapes. While the presence of an intraguild competitor may result in spatial avoidance and resource segregation in homogeneous landscapes, it is likely to increase the strength of apparent competition in more heterogeneous landscapes (Latham, Latham, Knopff, Hebblewhite, & Boutin, 2013). At Packsaddle, spatial heterogeneity offered increased hunting opportunities by increasing possible hunting habitats for both the Red‐tailed hawk and the Northern Harrier. Nevertheless it put both predators in competitive proximity possibly creating a system of trade‐offs between increased hunting areas and possible reduced energy intake.

Our study supports the growing body of evidence that local interactions among competitors may vary in strength across gradients of heterogeneity. We demonstrated that differences in fine‐scale habitat selection might be responsible for the realized niche segregation and overlap between the Red‐tailed hawk and the Northern Harrier along heterogeneity gradients. This could have important management implications at the WMAs where we conducted the research. For example, Packsaddle WMA focuses management on prescribed fire and other efforts to encourage growth of shinnery oak, and including the hybrid shinnery oak motte structure that can provide valuable cover for Northern Bobwhite (*Colinus virginianus*) quail, a species of conservation concern in the Great Plains (Carroll, Davis, Elmore, Fuhlendorf, & Thacker, 2015). Both Red‐tailed hawk and Northern Harrier are, however, facultative predators of Northern Bobwhite. Thus, Northern Bobwhite are potentially vulnerable to predation from two raptor species across much of Packsaddle WMA but the greater segregation of habitat selection at Beaver River results in much of the WMA leaving the quail vulnerable to just one of the predators at a time. Future research in this system could directly examine predator‐specific rates of predation on quail under different levels of heterogeneity. This could potentially lead to targeted management prescriptions that strike a balance between providing important thermal cover for Northern Bobwhite where they are also less exposed to predators.

## CONFLICT OF INTEREST

None declared.
