# Supplementary material for: Spatial heterogeneity and scale‐dependent habitat selection for two sympatric raptors in mixed‐grass prairie
Source: Ecol Evol. 2017 Jul 15;7(16):6559–69. doi: 10.1002/ece3.3182 (PMC5574806; doi:10.1002/ece3.3182)
Supplement: Supplementary file 1 [file ECE3-7-6559-s001.docx]

**Online supporting information**

**Tables**

Table S1: Summary details for models fitted to Northern Harrier (NOHA) and Red-tailed Hawk (RTHA) at Packsaddle Wildlife Movement Area, Oklahoma, USA, 2013–2015. Detection functions were defined using parametric key including half-normal (hn), hazard-rate (hr), and uniform (unif). Also shown are effective detection width (ESW), P-values for χ2 (Chi-p), and Kolmogorov–Smirnov (KS) goodness-of-fit tests.

| Species | Name | Key function | par | ΔAIC | AIC | ESW | Chi-p | K-S |
| --- | --- | --- | --- | --- | --- | --- | --- | --- |
| NOHA | observer x TOD | HR | 10 | 0.00 | 4350.34 | 189.72 | 7E-04 | 0.02 |
|  | observer | HR | 6 | 4.05 | 4354.40 | 201.85 | 9E-03 | 0.31 |
|  | observer | HN | 5 | 12.95 | 4363.29 | 201.54 | 2E-02 | 0.24 |
|  | observer x month | HR | 13 | 14.28 | 4364.62 | 198.83 | 3E-04 | 0.26 |
|  | observer x month | HN | 12 | 17.27 | 4367.61 | 196.38 | 5E-04 | 0.12 |
|  | month x TOD | HN | 12 | 51.13 | 4401.47 | 201.24 | 5E-04 | 0.05 |
|  | month | HN | 8 | 52.79 | 4403.13 | 204.73 | 4E-03 | 0.22 |
|  | month x TOD | HR | 13 | 55.22 | 4405.57 | 207.86 | 1E-03 | 0.14 |
|  | month | HR | 9 | 55.48 | 4405.82 | 215.26 | 1E-02 | 0.41 |
|  | TOD | NR | 6 | 67.36 | 4417.71 | 210.84 | 2E-02 | 0.10 |
|  | no covariate | HR | 2 | 70.28 | 4420.62 | 219.98 | 2E-01 | 0.29 |
|  | TOD | HN | 5 | 72.81 | 4423.15 | 216.44 | 1E-02 | 0.10 |
|  | observer x TOD | HR | 1 | 75.60 | 4425.95 | 219.80 | 3E-02 | 0.10 |
|  | no covariate | HN | 1 | 75.60 | 4425.95 | 219.80 | 3E-02 | 0.10 |
|  | no covariate | Unif | 2 | 76.26 | 4426.60 | 210.91 | 4E-02 | 0.18 |
| RTHA | observer | HR | 6 | 0.00 | 11058.07 | 240.28 | 1.0E-04 | 0.20 |
|  | TOD x observer | HR | 10 | 2.56 | 11060.63 | 232.94 | 4.9E-06 | 0.07 |
|  | month x observer | HR | 17 | 11.09 | 11069.16 | 237.26 | 0.0E+00 | 0.31 |
|  | time x observer | HN | 9 | 20.36 | 11078.43 | 233.45 | 8.9E-06 | 0.13 |
|  | observer | HN | 5 | 24.54 | 11082.61 | 235.36 | 7.6E-05 | 0.07 |
|  | month | HR | 13 | 24.74 | 11082.81 | 241.21 | 9.5E-07 | 0.29 |
|  | month x observer | HN | 16 | 32.48 | 11090.55 | 232.71 | 6.0E-08 | 0.17 |
|  | month | HN | 12 | 43.64 | 11101.71 | 235.48 | 1.1E-06 | 0.22 |
|  | TOD x month | HR | 16 | 46.58 | 11104.65 | 235.42 | 6.0E-08 | 0.29 |
|  | TOD x month | HN | 16 | 46.58 | 11104.65 | 235.42 | 6.0E-08 | 0.29 |
|  | TOD | HR | 6 | 50.51 | 11108.58 | 253.85 | 2.5E-04 | 0.48 |
|  | TOD | HN | 5 | 50.78 | 11108.85 | 239.26 | 5.9E-05 | 0.18 |
|  | no covariate | HR | 2 | 53.54 | 11111.61 | 255.75 | 1.5E-03 | 0.40 |
|  | no covariate | Unif | 1 | 58.10 | 11116.17 | 248.26 | 4.1E-04 | 0.21 |
|  | no covariate | HN | 1 | 60.23 | 11118.30 | 241.92 | 2.9E-04 | 0.12 |

*TOD = Time of day

Table S2: Summary details for models fitted to Northern Harrier (NOHA) and Red-tailed Hawk (RTHA) detection at Beaver River Wildlife Management Area, Oklahoma, USA, 2013–2015. Detection functions were defined using parametric key including half-normal (hn), hazard-rate (hr), and uniform (unif). Also shown are effective detection width (ESW), P-values for χ2 (Chi-p), and Kolmogorov–Smirnov (KS) goodness-of-fit tests.

| Site | Species | Name | Key function | par | ΔAIC | AIC | ESW | Chi-p | K-S |
| --- | --- | --- | --- | --- | --- | --- | --- | --- | --- |
| Beaver | NOHA | month | HN | 10 | 0.00 | 20.00 | 193.05 | 0.00 | 0.03 |
|  |  | month | HR | 11 | 2.00 | 22.00 | 203.87 | 0.00 | 0.03 |
|  |  | TOD x month | HN | 14 | 8.00 | 28.00 | 191.43 | 0.00 | 0.03 |
|  |  | observer x month | HN | 14 | 8.00 | 28.00 | 187.00 | 0.00 | 0.03 |
|  |  | observer | HR | 6 | 13991.09 | 14011.09 | 187.55 | 0.00 | 0.03 |
|  |  | observer x TOD | HR | 10 | 13992.55 | 14012.55 | 187.89 | 0.00 | 0.03 |
|  |  | observer x month | HR | 15 | 14001.51 | 14021.51 | 188.78 | 0.00 | 0.03 |
|  |  | observer | HN | 5 | 14041.04 | 14061.04 | 203.19 | 0.05 | 0.03 |
|  |  | observer x TOD | HN | 9 | 14041.53 | 14061.53 | 202.18 | 0.01 | 0.03 |
|  |  | TOD x month | HR | 15 | 14082.56 | 14102.56 | 205.33 | 0.00 | 0.03 |
|  |  | TOD | HR | 6 | 14114.70 | 14134.70 | 208.12 | 0.00 | 0.03 |
|  |  | TOD | HN | 5 | 14119.31 | 14139.31 | 212.02 | 0.03 | 0.03 |
|  |  | unif | Unif | 4 | 14126.60 | 14146.60 | 208.34 | 0.17 | 0.03 |
|  |  | HN | HN | 2 | 14127.49 | 14147.49 | 201.01 | 0.14 | 0.03 |
|  |  | HR | HR | 2 | 14134.90 | 14154.90 | 220.80 | 0.02 | 0.03 |
|  | RTHA | observer x month | HR | 18 | 0.00 | 16821.96 | 279.35 | 0.00 | 0.07 |
|  |  | month | HR | 13 | 9.87 | 16831.83 | 280.34 | 0.00 | 0.10 |
|  |  | month X TOD | HR | 17 | 11.42 | 16833.38 | 277.54 | 0.00 | 0.06 |
|  |  | observer x month | HN | 17 | 15.14 | 16837.10 | 248.54 | 0.00 | 0.00 |
|  |  | month | HN | 12 | 25.10 | 16847.06 | 251.04 | 0.00 | 0.00 |
|  |  | month x TOD | HN | 16 | 27.41 | 16849.37 | 250.29 | 0.00 | 0.00 |
|  |  | observer + TOD | HR | 11 | 44.57 | 16866.53 | 282.03 | 0.00 | 0.14 |
|  |  | observer | HN | 6 | 47.74 | 16869.70 | 254.45 | 0.00 | 0.00 |
|  |  | TOD | HR | 6 | 47.74 | 16869.70 | 254.45 | 0.00 | 0.00 |
|  |  | Observer + TOD | HN | 10 | 52.18 | 16874.14 | 253.91 | 0.00 | 0.00 |
|  |  | HN | HN | 2 | 86.89 | 16908.85 | 285.87 | 0.00 | 0.04 |
|  |  | HR | HN | 2 | 87.13 | 16909.09 | 290.57 | 0.00 | 0.08 |
|  |  | TOD | HR | 6 | 87.23 | 16909.19 | 293.10 | 0.00 | 0.09 |
|  |  | TOD | HN | 5 | 87.67 | 16909.63 | 258.99 | 0.00 | 0.00 |
|  |  | Unif | Unif | 3 | 89.20 | 16911.16 | 284.12 | 0.00 | 0.03 |

*TOD = Time of day

Table S3: Summary of results from GLMM resource selection models for Red-tailed Hawk (RTHA) and Northern Harrier (NOHA) at Beaver River Wildlife Management Area. Shown are the top 3 models or all nested models (> 3 nested models) with associated degrees of freedom (df), AICc, ΔAICc and Akaike model weights (Ѡi) collected at fine and broad scales.

| Scale (ha) | Species | model | df | AICc | ΔAICc | Ѡi |
| --- | --- | --- | --- | --- | --- | --- |
| **Fine** | NOHA | bare ground+grass+upland shrub+ riparian forest+sparse veg. | 5 | 884.60 | 0.00 | 0.59 |
| **(28. 27)** |  | bare ground+grass+riparian shrub+upland shrub+sparse veg.+ riparian forest | 6 | 886.70 | 2.11 | 0.21 |
|  |  | bare ground+grass+upland shrub+sparse veg | 6 | 887.60 | 2.99 | 0.13 |
|  | RTHA | bare ground+grass+riparian shrub+upland shrub+riparian forest | 7 | 1429.60 | 0.00 | 0.13 |
|  |  | grass+riparian shrub+upland shrub+riparian forest | 6 | 1429.80 | 0.16 | 0.12 |
|  |  | riparian shrub+upland shrub+riparian forest | 5 | 1430.10 | 0.50 | 0.10 |
|  |  | grass+riparian shrub+upland shrub | 6 | 1430.20 | 0.57 | 0.10 |
|  |  | bare ground+grass+riparian shrub+upland shrub | 5 | 1430.50 | 0.89 | 0.08 |
|  |  | bare ground+grass+upland shrub+riparian forest | 6 | 1430.60 | 0.96 | 0.08 |
|  |  | grass+upland shrub | 4 | 1431.00 | 1.35 | 0.07 |
|  |  | grass+upland shrub+riparian forest | 5 | 1431.30 | 1.72 | 0.06 |
| **Broad** |  | ***Vegetation variables*** |  |  |  |  |
| **(201.06)** | NOHA | sparse veg+upland shrub+riparian forest | 5 | 923.50 | 0.00 | 0.11 |
|  |  | sparse veg+grass+riparian forest | 5 | 924.00 | 0.50 | 0.09 |
|  |  | riparian forest | 3 | 924.10 | 0.59 | 0.08 |
|  |  | sparse veg+riparian forest | 4 | 924.20 | 0.62 | 0.08 |
|  |  | upland shrub+riparian forest | 4 | 924.60 | 1.03 | 0.07 |
|  |  | sparse veg+upland shrub+riparian forest+grass | 6 | 924.60 | 1.07 | 0.06 |
|  |  | grass+upland forest | 4 | 924.90 | 1.41 | 0.05 |
|  | RTHA | bare ground+riparian shrub+upland shrub+riparian forest | 8 | 1376.60 | 0.00 | 0.29 |
|  |  | bare ground+riparian shrub+upland shrub | 7 | 1377.70 | 1.07 | 0.17 |
|  |  | bare ground+grass+riparian shrub+upland shrub+riparian forest | 9 | 1378.10 | 1.47 | 0.14 |
|  |  | riparian shrub+upland shrub+riparian forest | 7 | 1378.50 | 1.88 | 0.11 |
|  |  | ***Distance variables*** |  |  |  |  |
|  | NOHA | dist.paved roads+dist.access roads+dist. oil pads+ dist. River | 5 | 473.60 | 0.00 | 1.00 |
|  |  | dist.paved roads+dist.access roads+dist. oil pads | 4 | 498.10 | 24.53 | 0.00 |
|  |  | dist.access roads+dist. oil pads+ dist. River | 4 | 498.90 | 25.27 | 0.00 |
|  | RTHA | dist.paved roads+dist.access roads+dist. oil pads+ dist. River | 5 | 419.90 | 0.00 | 0.644 |
|  |  | dist.paved roads+dist. oil pads+ dist. River | 4 | 421.10 | 1.18 | 0.356 |
|  |  | dist.access roads+dist. oil pads+ dist. River | 4 | 480.60 | 60.71 | 0.00 |
|  |  | ***Topographical variables*** |  |  |  |  |
|  | NOHA | slope | 2 | 929.90 | 0.00 | 0.751 |
|  |  | elevation | 2 | 935.70 | 5.74 | 0.043 |
|  |  | cosine aspect | 2 | 935.70 | 5.75 | 0.042 |
|  | RTHA | elevation^2^ | 2 | 1511.00 | 0.00 | 0.479 |
|  |  | elevation^2^+slope | 3 | 1512.30 | 1.33 | 0.246 |
|  |  | elevation+elevation^2^ | 3 | 1513.70 | 2.71 | 0.124 |

Table S4: Summary of results from GLMM resource selection models for the Red-tailed Hawk (RTHA) and the Northern Harrier (NOHA) at Packsaddle Wildlife Management Area Shown are the top 5 models associated degrees of freedom (df), AICc, ΔAICc and Akaike model weights (Ѡi) collected at fine and broad scales.

| Scale (ha) | Species | models | df | AIC_c_ | ΔAIC_c_ | Ѡ_i_ |
| --- | --- | --- | --- | --- | --- | --- |
| Small | RTHA | riparian forest | 2 | 702.90 | 0.00 | 0.77 |
|  |  | oil pads+ riparian forest | 3 | 706.58 | 3.68 | 0.12 |
|  |  | riparian forest+ riparian forest | 3 | 708.28 | 5.38 | 0.05 |
|  |  | upland forest | 2 | 710.55 | 7.66 | 0.02 |
|  |  | oil pads+upland forest+bare ground | 4 | 711.29 | 8.39 | 0.01 |
|  | NOHA | grass cover+upland forest | 3 | 533.88 | 0.00 | 0.35 |
|  |  | grass cover+upland forest+grass cover*upland forest | 4 | 533.98 | 0.10 | 0.34 |
|  |  | bare ground+riparian forest | 3 | 536.21 | 2.33 | 0.11 |
|  |  | Grass cover+upland forest+bare ground | 4 | 536.80 | 2.92 | 0.08 |
|  |  | grass cover | 2 | 538.28 | 4.40 | 0.04 |
| Broad |  | vegetation |  |  |  |  |
|  | RTHA | grass cover+riparian forest+upland forest | 3 | 717.20 | 0.00 | 0.99 |
|  |  | bare ground+oil pads+riparian forest | 4 | 727.42 | 10.22 | 0.01 |
|  |  | bare ground+grass cover+riparian forest+upland forest | 5 | 729.50 | 12.30 | 0.00 |
|  |  | bare ground+oil padr+riparian forest+upland forest | 5 | 732.02 | 14.82 | 0.00 |
|  |  | oil padr+riparian forest+upland forest+grass cover*oil pad | 6 | 734.77 | 17.57 | 0.00 |
|  | NOHA | grass cover+upland fores | 2 | 271.91 | 0.00 | 0.40 |
|  |  | grass cover | 2 | 273.51 | 1.60 | 0.18 |
|  |  | upland forest+oil pad | 3 | 273.78 | 1.87 | 0.16 |
|  |  | bare ground+oil pad | 3 | 273.96 | 2.06 | 0.14 |
|  |  | bare ground | 3 | 275.40 | 3.50 | 0.07 |
|  | NOHA | Distance variables |  |  |  |  |
|  |  | dist.access road+dist.paved road+dist.oil pads+dist.river | 4 | 256.61 | 0.00 | 0.27 |
|  |  | dist.access road+dist.paved road+dist.river | 5 | 256.96 | 0.35 | 0.22 |
|  |  | dist.access road+dist.river | 3 | 257.68 | 1.08 | 0.16 |
|  |  | dist.access road+dist.paved road+dist.oil pads | 4 | 258.65 | 2.04 | 0.10 |
|  |  | dist.paved road+dist.oil pads+dist.river | 4 | 258.85 | 2.24 | 0.09 |
|  |  |  |  |  |  |  |
|  | RTHA | dist.paved road | 2 | 564.70 | 0.00 | 0.30 |
|  |  | dist.paved road+dist.river | 3 | 564.80 | 0.09 | 0.29 |
|  |  | dist.paved road+dist.oil pads | 3 | 565.70 | 0.97 | 0.19 |
|  |  | dist.paved road+dist.oil pads+dist.river | 4 | 566.00 | 1.30 | 0.16 |
|  |  | dist.paved road+dist.access road+dist.oil pad | 4 | 571.14 | 6.39 | 0.01 |
|  | NOHA | Topographical variables |  |  |  |  |
|  |  | elevation2+slope | 3 | 258.26 | 0.00 | 0.39 |
|  |  | slope | 2 | 259.04 | 0.78 | 0.26 |
|  |  | elevation2 | 2 | 260.01 | 1.76 | 0.16 |
|  |  | sine aspect+slope+sine aspect*slope | 4 | 262.43 | 4.18 | 0.05 |
|  |  | sin aspect+slope | 4 | 263.11 | 4.86 | 0.03 |
|  | RTHA | slope | 2 | 711.09 | 0.00 | 0.55 |
|  |  | cosine aspect | 2 | 714.44 | 3.35 | 0.10 |
|  |  | sine aspect | 2 | 715.03 | 3.94 | 0.08 |
|  |  | elevation | 2 | 715.08 | 3.99 | 0.08 |
|  |  | elevation2 | 2 | 715.16 | 4.08 | 0.07 |

Table S5: Outlying Mean Index (OMI) analysis between Red-tailed Hawk (RTHA) and Northern Harrier (NOHA) to a suite of environmental variables at Beaver River and Packsaddle Wildlife Management Areas. Inertia = variance or weighted sum of squared distances to the origin of the environmental axes; OMI = outlying mean index (marginality); Tol = tolerance index, which represents niche breadth of the two predators across the measured environmental variables; RTol = residual tolerance. Italicized terms represent the percentages of variability corresponding to a specific statistic. P = frequency based on number of random permutations (out of 10,000) that yielded a higher value than the observed outlying mean index (P ≤ 0.05 indicates a significant influence of the environmental variables for a species).

| Site | Species | inertia | OMI | Tol | *Rtol* | *omi* | *tol* | *rtol* | P-value |
| --- | --- | --- | --- | --- | --- | --- | --- | --- | --- |
| Beaver River | RTHA | 11.71 | 0.32 | 2.47 | 8.92 | 2.70 | 21.10 | 76.20 | <0.001 |
|  | NOHA | 10.28 | 0.10 | 1.27 | 8.91 | 1.00 | 12.40 | 86.60 | 0.020 |
| Packsaddle | RTHA | 8.54 | 0.02 | 1.35 | 7.17 | 0.20 | 15.90 | 83.90 | 0.360 |
|  | NOHA | 6.89 | 0.18 | 0.79 | 5.93 | 2.50 | 11.40 | 86.10 | 0.170 |

**Figures**

Figure S1: Hazard rate detection function plots showing the detection probabilities of four individual observers for Red-tailed Hawk (a), Northern Harrier (b) at Packsaddle WMA, and Red-tailed Hawk (c), Northern Harrier (d) at Beaver River WMA in Oklahoma, USA, 2013–2015.

Figure S1
